# Digital Health Literacy and Web-Based Health Information-Seeking Behaviors in the Saudi Arabian Population

**DOI:** 10.7759/cureus.51125

**Published:** 2023-12-26

**Authors:** Anas Alhur, Afrah Alhur, Muteb Alshammari, Arwa Alhur, Wafa Bin Shamlan, Muhannad Alqahtani, Samia Alhabsi, Raheeq Hassan, Ebtehal Baawadh, Shahad Alahmari, Noura Alshahrani, Rana Alwadae, Esra Abdalla, Hadeel Abuali, Malath Alshahrani

**Affiliations:** 1 Department of Health Informatics, University of Hail, Hail, SAU; 2 Department of Clinical Nutrition, University of Hail, Hail, SAU; 3 Department of Psychology, University of Hail, Hail, SAU; 4 Department of Pharmacy, Al Murjan Hospital, Jeddah, SAU; 5 Department of Pharmacy, Ministry of Health, Riyadh, SAU; 6 Faculty of Medicine, University of Khartoum, Sudan, SDN; 7 Department of Health Administration, Saudi Electronic University, Riyadh, SAU; 8 Department of Pharmacy, King Khalid University, Asir, SAU; 9 Department of Radiology, Ministry of Health, Riyadh, SAU; 10 Faculty of Medicine, National Ribat University, Sudan, SDN; 11 Department of Public Health, ‏King Khalid University, Abha, SAU

**Keywords:** information sources, digital skills, online health information, web-based health information seeking, digital health literacy

## Abstract

Introduction

In the evolving landscape of healthcare, the emergence of digital technologies has brought digital health literacy to the forefront. This is especially pertinent given the vast amount of health information available online and the diverse capabilities of individuals to effectively use this resource. Focusing on the Saudi Arabian context, where digital health practices are increasingly integrated into daily life, our study aims to investigate the competencies in digital health literacy and the patterns of online health information seeking among the Saudi population.

Methods

A quantitative research design was adopted for this investigation. Data were collected through online surveys from a diverse cohort of 2,184 Saudi residents, all above the age of 18 years. The survey was designed to evaluate the participants' ability to find, understand, and use health information obtained from digital platforms.

Results

Analysis indicates that 63% of the population is proficient in using search engines for health information. However, 37% face challenges in formulating precise health-related inquiries. Additionally, the study identified significant variations in digital health literacy across different genders and age groups, with younger participants generally showing higher literacy levels.

Conclusion

The results of our study highlight the critical need for specialized educational initiatives in Saudi Arabia aimed at bolstering digital health literacy. This is particularly essential in bridging the gaps observed in different age groups and between genders. By enhancing these key competencies, we can significantly empower individuals to make well-informed health decisions. Such advancements are instrumental in nurturing a society that is both more informed and proficient in managing health-related information in a digital context.

## Introduction

In an era marked by rapid technological advancements, the fusion of healthcare and digital technology has revolutionized the way individuals access, comprehend, and use health information. This transformation is embodied by the concept of digital health literacy, often interchangeably referred to as eHealth literacy. Digital health literacy encompasses a range of competencies essential for effectively navigating, comprehending, evaluating, and applying health-related information obtained from electronic sources. As healthcare increasingly integrates digital solutions, understanding the intricacies of digital health literacy becomes paramount.

The inception of the term "eHealth literacy" by Norman and Skinner in 2006 highlighted its pivotal role in health information acquisition and application [1]. Neter and Brainin underscored the enduring significance of digital health literacy in today's digitally-driven age, especially in fostering patient engagement and informed decision-making [2]. Paakkari and Paakkari delved into the realm of interactive literacy, which pertains to adept digital communication between healthcare stakeholders [3]. Chung and Nahm emphasized the importance of critical literacy as a cornerstone for making informed health decisions [4]. Gilstad drew attention to foundational skills such as proficiently using search engines and navigating complex health websites [5]. Diviani et al. underscored the paramount importance of sourcing accurate and reliable health information from the vast digital landscape [6]. Moreover, in a study focusing on Hail City residents in Saudi Arabia, it was found that a significant portion of participants predominantly relied on search engines (48.3%) and official websites (27.9%) as their main sources for obtaining health information [7].

Digital health literacy, however, is not a one-size-fits-all concept. Demographics play a significant role in shaping an individual's proficiency in navigating the digital health landscape, as noted by Mackert et al. [8]. Motivation and self-confidence have been linked to the extent of engagement with digital health resources, as observed by Norgaard et al. [9]. The repercussions of low digital health literacy, as cautioned by Mitsutake et al., underscore the critical need to understand and enhance this competence [10]. Furthermore, experts such as Mackert et al. and Zarcadoolas et al. have advocated for educational interventions and the creation of user-friendly health platforms to bridge the digital health literacy gap [8,11].

The ascendancy of the internet has dramatically transformed the landscape of health information access and seeking behaviors. Multiple research studies conducted in the Kingdom of Saudi Arabia (KSA) have revealed that a significant portion of the Saudi population is technologically connected [11-19]. Moreover, Fox et al. found that a majority of internet users globally have sought health-related information online [20]. Powell et al. even established a link between online health searches and the onset of symptoms or impending medical visits [21]. Lambert and Loiselle have identified primary motivations driving individuals to seek health information online [22]. However, this proliferation of health information on the internet has also raised concerns about its quality and reliability, as pointed out by researchers such as Eysenbach and Köhler [23] and Diviani et al. [6]. Additionally, demographic factors have been shown to influence online health information-seeking behaviors, as observed by Neter and Brainin [2].

In light of these developments and the unique socio-cultural context of Saudi Arabia, this research aims to investigate the state of digital health literacy competence and web-based health information-seeking behaviors among the Saudi population. The overarching objectives of this study include assessing the current levels of digital health literacy, identifying demographic factors that influence digital health literacy, exploring motivations and behaviors related to online health information seeking, evaluating the sources and perceptions of online health information quality, and uncovering gaps in digital health literacy skills. By achieving these objectives, this research aims to provide valuable insights and recommendations for enhancing digital health literacy and improving the accessibility of credible health information online. Ultimately, this study contributes to a deeper understanding of how digital health literacy impacts health information use and decision-making among the Saudi population, with far-reaching implications for future interventions and healthcare outcomes.

## Materials and methods

Research framework

This study operates within a quantitative research paradigm, focusing on digital health competencies and online search behaviors among individuals in Saudi Arabia. This method provides a comprehensive framework for examining current trends in this area.

Sampling strategy

To ensure a representative sample of the resident Saudi Arabian population, we employed a random sampling method. Our inclusion criteria required participants to be 18 years or older and residents of Saudi Arabia, while individuals below the age of 18 were excluded. Notably, 74.6% of our respondents resided in urban areas, while the remaining participants were from rural locations.

Minimum sample size calculation

We determined the minimum sample size using a standard formula that takes into account population size, expected frequency, margin of error, and confidence level. This calculation was instrumental in ensuring the statistical power needed for the validation of our research findings.

Data collection

Data collection took place through online surveys, which were available from September 3 to November 22, 2023. The questionnaire was presented in both Arabic and English languages and remained accessible for an extended period to facilitate responses. We distributed the survey across various platforms, including health-focused websites and social media, to reach a diverse demographic.

Instrumentation

Our questionnaire, built upon established constructs, was customized to align with the specific objectives of this study. Prior to full-scale data collection, a pilot test was conducted to validate the questionnaire and enhance its accuracy.

Analytical techniques

Following data collection, we performed statistical analyses using SPSS Version 26 (IBM Corp. Armonk, NY). These analyses encompassed the computation of means, modes, medians, and standard deviations to reveal significant trends and relationships within the data.

Ethical adherence

Ethical approval was obtained from the Hail Health Cluster Ethical Approval Committee (No. 2023-82). Participants provided implied consent by initiating the survey online.

Validation of the questionnaire

Our questionnaire underwent a rigorous validation process, including expert reviews and a pilot study, to ensure its relevance and reliability for this research endeavor.

## Results

**Figure 1:**
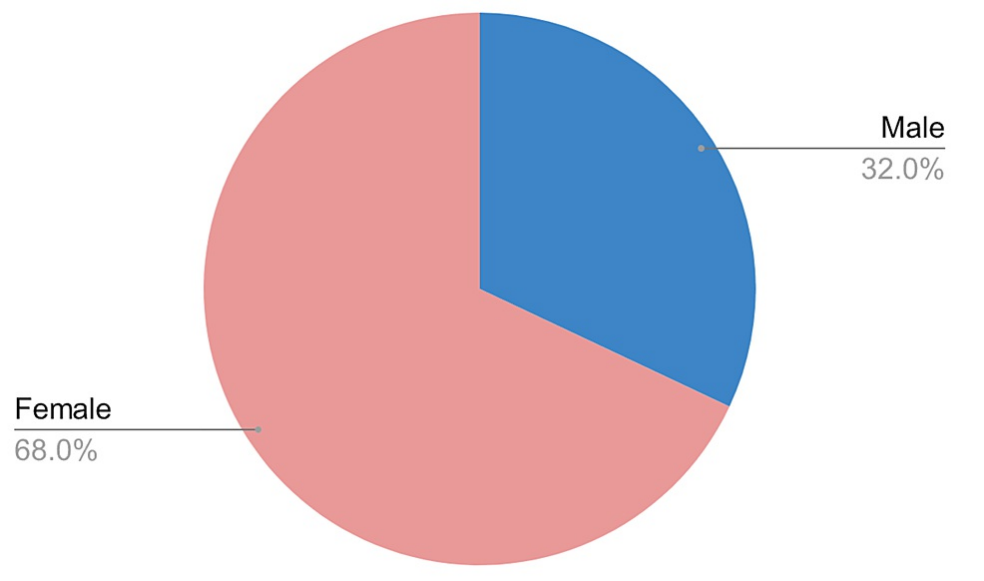
Respondents' gender distribution (N=2,184)

**Figure 2:**
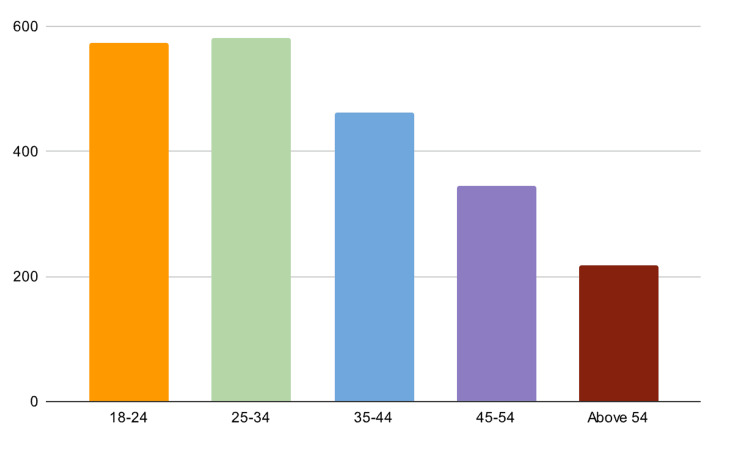
Respondents' age distribution (N=2,184)

From the total respondents approached (N=2,184), the vast majority (98.0%) agreed to participate in the study, with only a tiny fraction (0.7%) declining (Table 1). In terms of "gender and age distribution," the survey had a total of 2,212 respondents. Among them, 708 were male, making up 32.0% of the total, while 1,504 were female, accounting for 68.0% (Figure 1). The age distribution was as follows: 574 respondents were between 18 and 24 years of age, making up 25.9% of the total (Figure 2). Overall, 582 respondents were between 25 and 34 years of age, accounting for 26.3%, 462 respondents were between 35 and 44 years of age, accounting for 20.9%, and 344 respondents were between 45 and 54 years of age, accounting for 15.6%. Finally, 218 respondents were above the age of 54 years, accounting for 9.9% of the total. When accounting for missing data, which amounted to 32, the grand total of participants remained at 2,212.

**Table 1:** Participation agreement

Response	Frequency (N)	Percentage (%)
Agree	2,168	98
Disagree	16	0.7
Total	2,212	100

In our survey, we sought to quantitatively assess the proficiency of respondents in navigating online health information. To achieve this, we measured the ease with which participants could perform specific tasks. These tasks included searching for health-related information, using correct medical terms, identifying accurate health information, formulating health-related inquiries, and assessing the reliability of the information found. Instead of using subjective terms such as “very easy” and “easy,” we analyzed the frequency of successful task completion and the average time taken for each task. This method provided a more objective measure of digital health literacy. Our results noted that a significant proportion of respondents could successfully locate health information within a brief time frame, suggesting a higher degree of proficiency in this aspect. Conversely, a considerable number of participants took longer to discern the reliability of health information, indicating a potential area for educational focus. 

The participants were also asked about their use of online health information, and only 32.4% reported the use of online resources and digital health information for commercial purposes. Also, 42.0% of the respondents found online health information effective for personal use, 1% perceived the reliability of online health information as sound, 51.9% found the health information on the internet to be consistent, and 35.9% used online health information for making health-related decisions.

Our study reveals interesting insights into how participants perceive online health information (Table 2). Notably, 32.4% (716 individuals) demonstrated their ability to recognize when online health information is commercially driven. However, 25.0% (554 individuals) struggled to make this distinction, and a significant 42.6% (942 individuals) were unsure about identifying such commercial motives.

**Table 2:** Ease and difficulty of online health information search and assessment

Item	Very easy (%)	Easy (%)	Difficult (%)	Very difficult (%)	Mean ± standard deviation
Ease of online health searches	598 (27%)	1,138 (51.4%)	409 (18.5%)	67 (3%)	1.98 ± 0.759
Using correct health-related terms	521 (23.6%)	1,231 (55.7%)	404 (18.3%)	56 (2.5%)	2 ± 0.721
Locating precise health information	441 (19.9%)	1,115 (50.4%)	560 (25.3%)	96 (4.3%)	2.14 ± 0.779
Formulating health-related questions	400 (18.1%)	1,103 (49.9%)	617 (27.9%)	92 (4.2%)	2.18 ± 0.77
Articulating health opinions in writing	400 (18.1%)	1,084 (49%)	625 (28.3%)	103 (4.7%)	2.19 ± 0.782
Ensuring comprehension by recipients	384 (17.4%)	1,028 (46.5%)	702 (31.7%)	98 (4.4%)	2.23 ± 0.784
Deciding information reliability	289 (13.1%)	672 (30.4%)	850 (38.4%)	401 (18.1%)	2.62 ± 0.928
Identifying commercial interests	331 (15%)	747 (33.8%)	820 (37.1%)	314 (14.2%)	2.4 ± 0.913
Deciding information applicability	329 (14.9%)	1,029 (46.5%)	705 (31.9%)	149 (6.7%)	2.3 ± 0.803
Applying information in daily life	335 (15.1%)	1,080 (48.8%)	658 (29.7%)	139 (6.3%)	2.27 ± 0.792
Making health decisions based on info	329 (14.9%)	1,181 (53.4%)	587 (26.5%)	115 (5.2%)	2.22 ± 0.757

When it comes to using online health information for personal needs, 42.0% (929 individuals) found it effective, while 23.3% (515 individuals) faced challenges in finding information suitable for their needs. The ambivalence of 34.7% (768 individuals) toward the efficacy of this information points to the need for further analysis in this area.

Regarding the reliability of online health information, participants' opinions were split: 30.1% (665 individuals) viewed the information as trustworthy, whereas 32.9% (727 individuals) had doubts about its credibility. Notably, 37.1% (820 individuals) remained undecided, underscoring diverse opinions on the trustworthiness of online health resources. Additionally, while more than half of the respondents (51.9% or 1,147 individuals) generally trusted the health information found online, 19.6% (433 individuals) reported encountering inconsistent information, and 28.6% (632 individuals) were unsure of its reliability.

In terms of decision-making, 35.9% (794 individuals) actively used online health information to make health-related decisions, contrasting with 29.0% (642 individuals) who did not rely on such data. A considerable portion, 35.1% (776 individuals), had not formed a definitive opinion on using online health information for decision-making, highlighting varied confidence levels among users in leveraging digital health resources.

**Figure 3:**
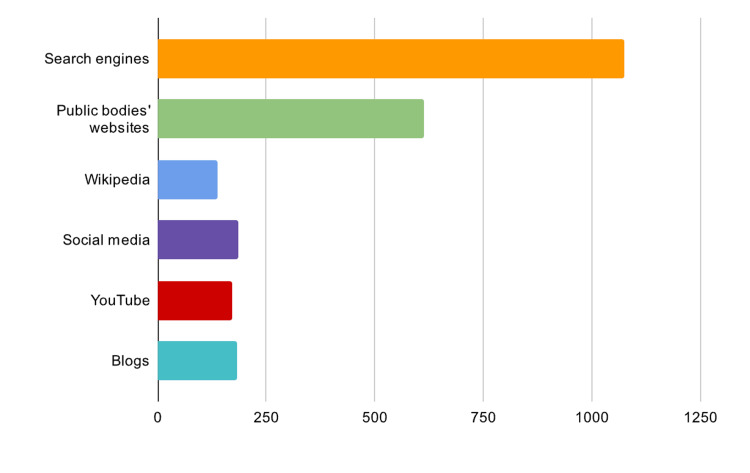
Sources used for obtaining health-related information

The survey revealed a diverse range of sources utilized by respondents for health-related information. The predominant sources, as indicated by 48.6% of participants, were search engines such as Google, Bing, and Yahoo, highlighting their central role in information dissemination. Following this, websites of public bodies, including RKI, BZgA, and various ministries of health, were identified as the second most frequented resource, used by 27.7% of respondents. This underscores the significant reliance on official and authoritative sources for health information. The bar graph in (Figure 3) illustrates these findings, clearly delineating the proportion of respondents favoring each source.

A full list of the survey questions referenced in the study can be found in Appendix A, Appendix B, and Appendix C, providing a context for the responses analyzed.

## Discussion

Our study reveals intriguing insights into the digital health literacy competence of the Saudi Arabian population. A significant majority of respondents found it easy to search for health-related information online. However, when it comes to assessing the reliability of the information, a considerable number found it challenging. The observation that low digital health literacy can result in poor health outcomes due to misinformation or misinterpretation of health information is well-documented in the literature [8]. A study also supports the observations made in the study by Fox et al. [20], in which it was noted that age, gender, education, and socio-economic status significantly determine digital health literacy levels. In our sample, females were more represented than males, and the age group of 25-34 years was the most active, which may indicate a higher level of digital health literacy among these groups.

The study also delved into the web-based health information-seeking behaviors of the Saudi Arabian population. Search engines were the most commonly used source for obtaining health-related information, followed by websites of public bodies. This finding resonates with the findings in the study by Powell et al. [21]. However, our study also found that a sizable percentage of respondents were unsure about the reliability and consistency of online health information. This uncertainty corroborates the concerns raised in other studies [22-24] about the quality and reliability of health information online.

Our study's findings are in line with the existing literature but offer a unique insight into the Saudi Arabian population. For instance, the high percentage of respondents leveraging online health information for commercial benefit and personal use supports the importance of digital health literacy highlighted in the study by Neter and Brainin [2]. The uncertainty about the reliability and consistency of online information in our study also aligns with the concerns raised in the study by Diviani et al. [6] about the need for help in differentiating between evidence-based information and anecdotal experiences.

Regarding the study's implications, our findings underscore the importance of digital health literacy interventions and user-friendly healthcare platforms. Specifically, the study suggests a need for educational initiatives aimed at enhancing digital health literacy among the Saudi Arabian population [9]. Additionally, the healthcare sector should prioritize the development of user-friendly digital platforms that accommodate individuals with varying levels of literacy [10]. Furthermore, our research highlights the potential for future studies to explore digital health literacy across different demographic groups in Saudi Arabia and to develop effective tools and methods for enhancing individuals' proficiency in digital health technology.

It is important to acknowledge some limitations in our study. We used online surveys, which could bias the sample toward those comfortable with digital tools. Self-reported data may lead to participants giving socially desirable answers rather than reflecting their actual behaviors. Our cross-sectional design provides a snapshot, missing potential changes over time. The study's generalizability might be limited to specific subgroups within Saudi Arabia. We did not deeply explore language and cultural factors. We mainly assessed digital health literacy for health information seeking, not broader digital skills or direct health outcomes. Qualitative data were not collected, limiting insights into participant motivations. Our questionnaire, while well-designed, may not cover all aspects of digital health literacy. Some participants did not complete the survey, which could introduce non-response bias. Finally, we briefly addressed the ability to discern online health information for commercial purposes without exploring this aspect in-depth. These limitations call for cautious interpretation and suggest areas for further research into digital health literacy and online health information-seeking behaviors in Saudi Arabia.

## Conclusions

Our exploration of digital health literacy and online health information-seeking behaviors in Saudi Arabia offers valuable insights. The high participation rate reflects strong engagement with digital health topics in the population. While there is a general ease in searching for health information online, challenges persist in assessing reliability.

Demographically, the predominance of females and the age group of 25-34 years in our survey results may indicate a trend toward younger individuals who are potentially more digitally literate and active in seeking health information. The data reveal a nuanced landscape, with some individuals comfortable using health information for personal purposes while others struggle to assess credibility.

The preference for search engines and official websites highlights the need for accuracy and reliability in health information. Enhancing the availability of credible health sources and making them accessible to the public is crucial for ensuring trustworthy online health information. Policymakers, educators, and developers should employ a comprehensive approach addressing accessibility, reliability, digital health literacy education, and critical evaluation skills to empower informed health decision-making in the digital era.

## Figures and Tables

**Figure 4:**
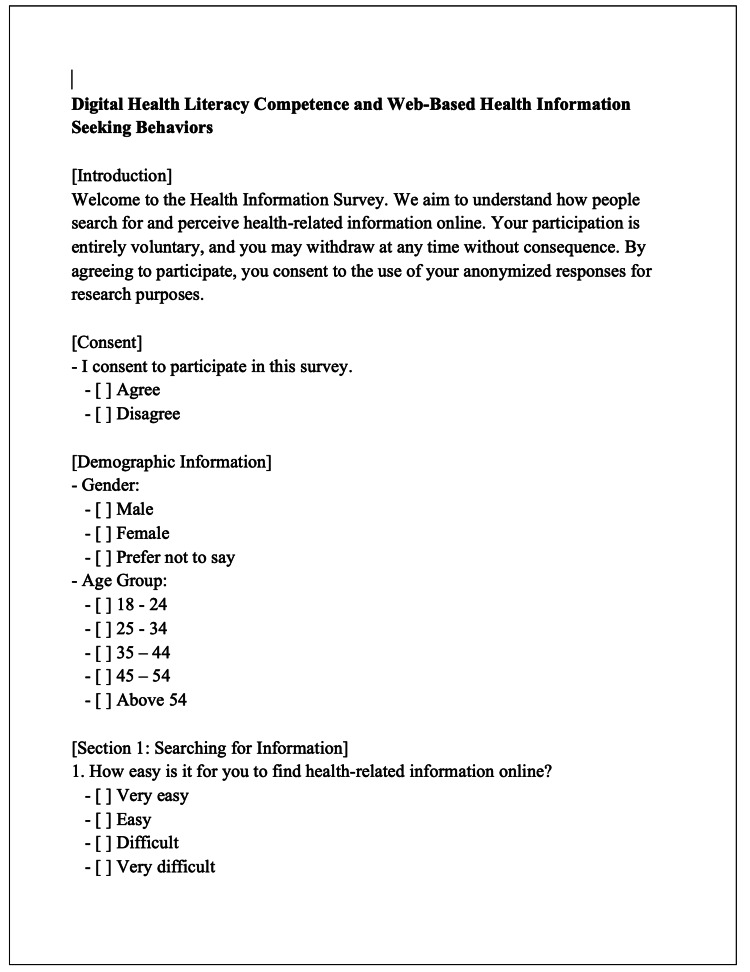
Digital health literacy competence and web-based health information-seeking behaviors.

**Figure 5:**
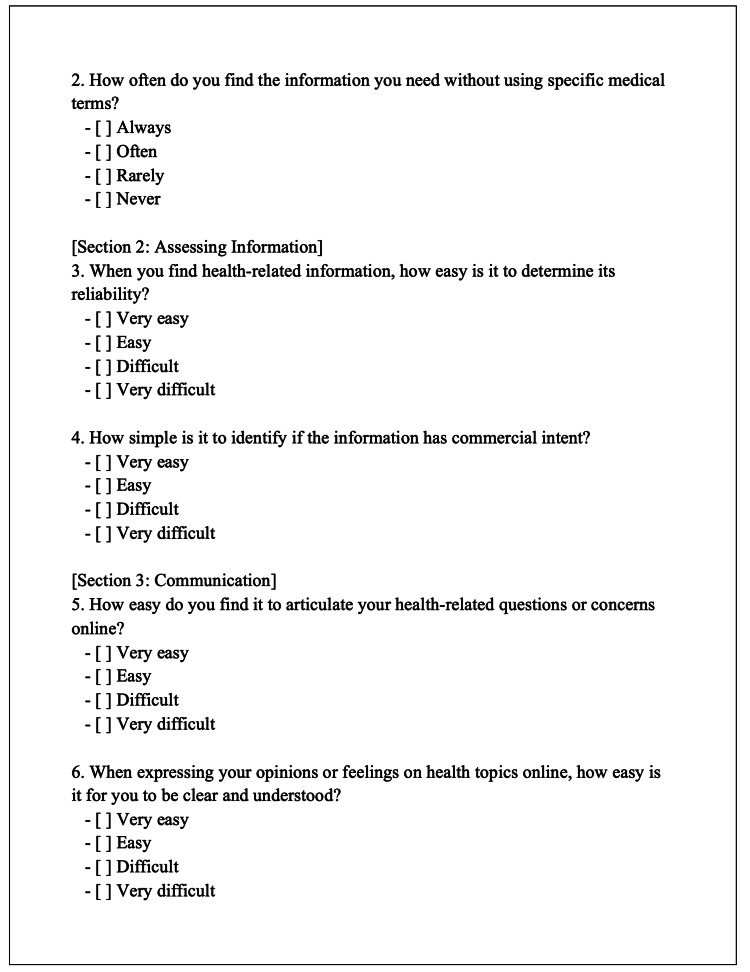
Digital health literacy competence and web-based health information-seeking behaviors.

**Figure 6:**
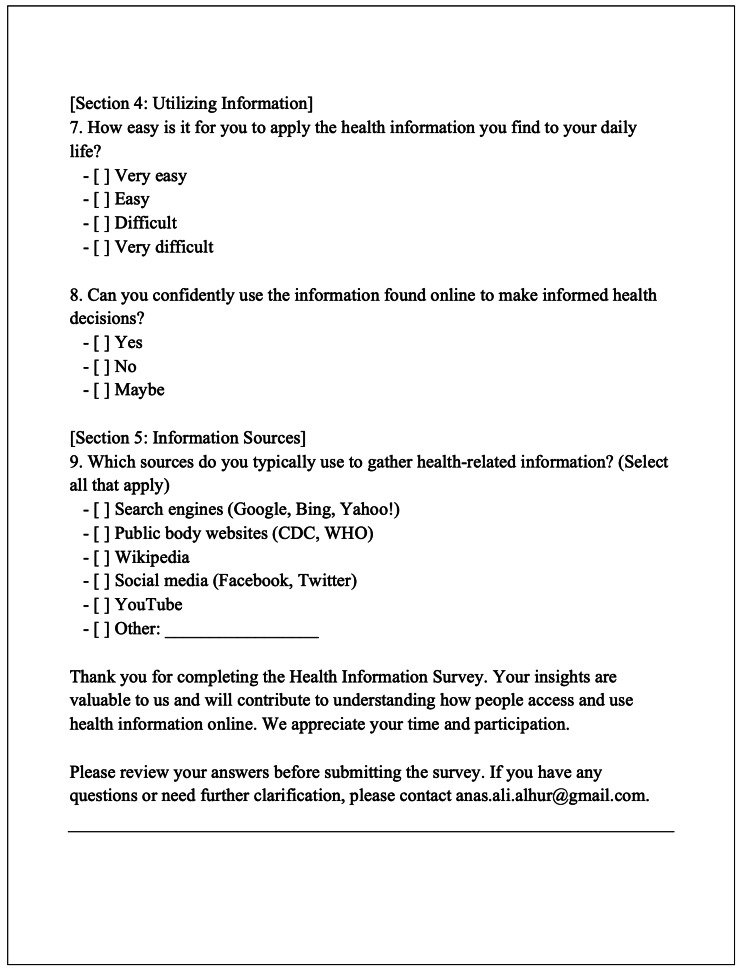
Digital health literacy competence and web-based health information-seeking behaviors.

## Appendices

Appendix A 

Appendix B 

Appendix C 
